# Assisted assembly: how to improve a *de novo *genome assembly by using related species

**DOI:** 10.1186/gb-2009-10-8-r88

**Published:** 2009-08-27

**Authors:** Sante Gnerre, Eric S Lander, Kerstin Lindblad-Toh, David B Jaffe

**Affiliations:** 1 Broad Institute of Harvard and MIT, Cambridge Center, Cambridge, Massachusetts 02142, USA; 2 Department of Medical Biochemistry and Microbiology, Uppsala University, Husarg.3, Uppsala 751 23, Sweden

## Abstract

We describe a new assembly algorithm, where a genome assembly with low sequence coverage, either throughout the genome or locally, due to cloning bias, is considerably improved through an assisting process via a related genome. We show that the information provided by aligning the whole-genome shotgun reads of the target against a reference genome can be used to substantially improve the quality of the resulting assembly.

## Abstract 2

A method is described for improving low sequence coverage genome assemblies

## Background

How completely one can reconstruct a genome sequence from whole-genome shotgun (WGS) reads depends on the depth of sequence coverage generated [1]. Additionally, longer reads and better base quality in reads provides more information and, therefore, allows any assembler to perform a better task, resulting in both the generation of bigger contigs/scaffolds and improvements in the quality of the assembly. The genomes of many species, including the mammals *Mus musculus *[2], *Canis familiaris *[3], and *Monodelphis domestica *[4], have been assembled from Sanger-chemistry WGS reads at, respectively, 6.1×, 7.6×, and 6.7× coverage, yielding drafts that represent nearly all of the genomes' euchromatic parts. These drafts are of high quality, and although imperfect, have served as references for the community.

However, at times, the cost of genome sequencing or the biological properties of a genome sequence will force a genome to be sequenced at lower coverage. Since mammalian genomes are large, cost was a major factor when, in 2004, the idea was conceived to annotate the human genome using the genome sequence of many mammals [5]. A lower coverage of the genome was then considered since, theoretically, at 2× coverage 1 - *e*^-2 ^≈ 86% of the genome is represented [1].

When theoretically considering the challenge behind low coverage assembly, we note that low coverage (either global or local) makes the assembly problem much harder to deal with, since it affects our capability of both distinguishing true from false read-read alignments and building a list of confirmed non-chimeric read pair links. Since an important step of the assembly process is to generate a set of read-read alignments, errors introduced in this step will have a major effect on the final product. If we somehow could generate only perfect data in this step (that is, the set of all and only the 'true' alignments, where 'true' means that two reads align if, and only if, they come from overlapping regions in the genome), then we could produce the optimal assembly of the sequence data. In general, however, we are not even close to the 'perfect' set, and we end up with both missing alignments (true alignments that are not detected), and with 'false' alignments (alignments of reads that actually belong to different regions of the genome). In addition, poor sequence quality, polymorphism and repetitiveness are reasons why true alignments may not be detected.

In principle, one could overcome this problem by introducing a method whereby low-coverage *de novo *assemblies may be improved via assistance from genome sequences of related species. If two species are very closely related, the problem is trivial since the overall genome structure is similar and read-read alignments to the related species will give the true position of reads also in the novel genome. However, in many cases no very similar genome exists as a template. As genomes become more diverged, two problems arise: reads may be more difficult to accurately align to the reference genome and biological differences in genome structure (that is, conserved synteny breakpoints, repeat insertions, and segmental duplications) may mean that the read-read placements on the reference are not reflective of the novel genome sequence. In terms of read placement, Margulies and co-workers established that using the BLASTZ algorithm [6] aligns reads reliably when the genomes diverge by up to approximately 0.45 substitutions per site. In addition, increased divergence usually correlates with increased amounts of genomic rearrangement.

We therefore conceived an assisted assembly method that works by reinforcing information that is already present in the reads. For example, consider two contigs connected by a single read pair. Because a small fraction (perhaps approximately 1%) of read pairs are chimeric - that is, result from a random ligation in the library construction process - joining the contigs would carry a roughly 1% risk of introducing a false join into the assembly. Now suppose both reads of the pair align consistently to a related genome. Because the odds that a chimeric read pair would align consistently is extremely low, we can safely join the contigs. Similarly, other information in a low-coverage data set may be suitably leveraged. We first tested this approach on the cat genome [7].

Here we describe the assisted assembly algorithms in detail, then test them on a low-coverage subset of a previously assembled high-coverage data set (*C. familiaris*), so that we can rigorously assess the effect of assistance on assembly accuracy, continuity and completeness. We then apply the method to several low-coverage mammals and the 8× *Plasmodium falciparum *HB3 assembly, which, due to cloning bias, is reduced to 2× or less over 15% of the genome [8]. The assisted assembly method gives marked improvements in all cases.

The source code for the assisted assembly algorithms and the assemblies themselves are available online [9].

### Assisted assembly algorithm

**Figure 1:**
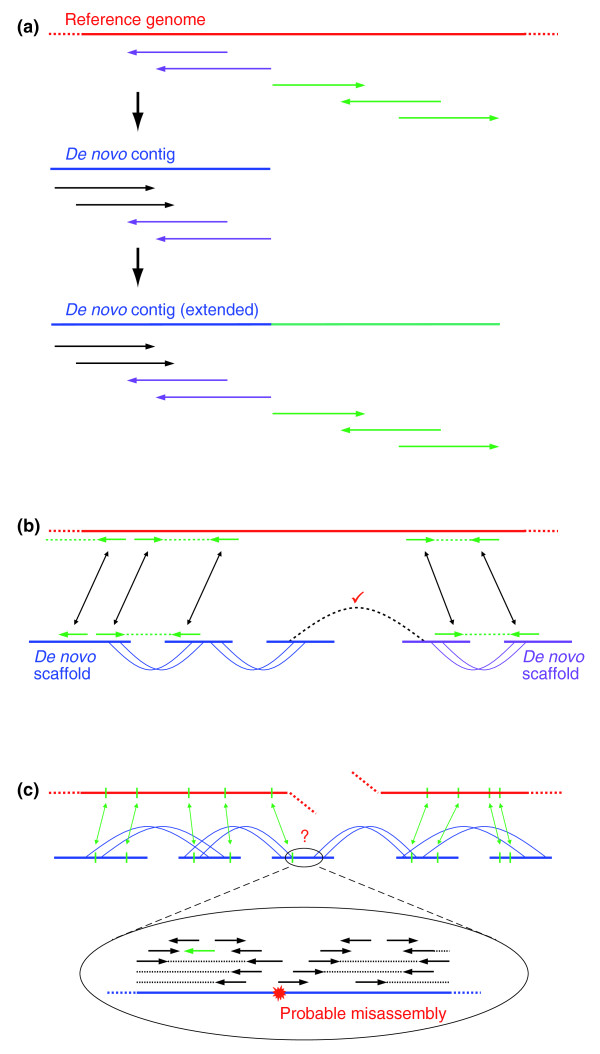
**Assisted assembly principle**. **(a) **In this example, five reads align uniquely to the reference genome, and the two leftmost of these (purple) also appear as the two rightmost reads in an existing *de novo *contig. We can then extend the *de novo *contig by using the three unassembled reads (green), even if there is no supporting linking evidence (in general, ARACHNE requires a read to be linked to the contig it overlaps before using it to extend the contig). **(b) **Two scaffolds (blue and purple) are mapped and oriented on the reference genome by the trusted green reads. Furthermore, the two scaffolds are joined by a single link (black dotted line), although this is not trusted *per se*. The ARACHNE scaffolding algorithm would not normally join the two scaffolds; however, in this case the separation of the two scaffolds implied by the link is consistent with the separation implied by the mapping on the reference genome, and we thus implicitly validate the black dotted link and join the two scaffolds. **(c) **Trusted read placements anchor portions of a single scaffold onto two distant parts of the reference genome, suggesting either a *bona fide *syntenic break or a misassembly. To test for the latter, the contested region on the scaffold is subject to a stringent test for misassembly, and broken if it fails. The same level of stringency of misassembly testing could not be applied to the entire assembly because, at low coverage, there would be too many false positives.

The assisted assembly process starts by simultaneously building a *de novo *assembly from the reads and by aligning the same reads to one or more related genomes. These alignments provide proximity relationships between the reads, which then seed changes to the assembly - for example, by adding in reads that had not been previously assembled. In the simplest case, a read has not been placed in a contig because its overlap with the contig is short. Now, with the additional evidence provided by cross-species proximity, the read can be placed with sufficient confidence. Similarly, alignment of a read pair to a related genome can validate the soundness of the read pair - virtually guaranteeing that it is not a chimera - thus allowing for a single read pair to join two scaffolds in the assembly. Once the initial assist has been performed, the algorithm iteratively carries out a series of standard assembly steps, such as adding in mate pairs, which can improve the quality of the assembly. This process may even correct errors introduced by the assistance process itself. Below and in Figure 1 we describe the key components of the assisted assembly algorithm.

#### Placing reads on a reference genome

Reads are separately aligned to the reference sequence for each related species. These alignments are local: a read is not required to align from end to end. This allows for reads to be placed in spite of evolutionary events, such as insertion of transposable elements, which are large relative to the read length. Reads may be placed multiply. Thus, if a region in the sample species' genome has been duplicated in the reference species, we can still use the related species to improve the assembly of the region.

#### Grouping reads (building proto-contigs)

For each read placement, we infer the read's start and stop points on the related genome, even if the placement does not extend from end to end. We then group read placements by continuity: we put reads together so long as their inferred start/stop intervals on the related genome overlap by at least one base. This overlap threshold is somewhat arbitrary: for purposes of grouping it could be increased or even made negative without conceptually altering the method.

#### Enlarging contigs

The reads in the groups are now used to enlarge the preexisting *de novo *assembly contigs (Figure 1a) and, in some cases, to start new contigs. To do this, we attempt to assign each group to a contig, by first finding all contigs that the group shares reads with. If there is one contig, we assign the group to that contig. If there are two contigs, as would happen if the group bridged a gap between them, we assign the group to the contig that it shares the most reads with. If there are more than two contigs, we do not assign the group. If there are no contigs, we extract one read from the group, call it a new contig, and assign the group to this new contig. Supposing that the group is assigned to a contig, we then take all the reads from the group that are not already in the contig, and align the reads one by one to the contig. If there is an end-to-end alignment between the read and the contig of at least a minimum length (24 nucleotides), the read is placed in the contig and the contig is modified if appropriate (for example adding bases on one end).

#### Joining scaffolds

In a *de novo *assembly, single read pair links cannot be used to join scaffolds, because even with a low rate of chimerism (for example, 1%) in libraries, there would still be too many incorrect joins. Given an assisting genome, however, we can define a single link as 'trusted' if it has a valid and unique alignment to the reference genome, and then use such single trusted links to join scaffolds. Allowing trusted links to join scaffolds would work - but inefficiently - because in practice only a fraction of the links are actually trusted. Instead, we first use the trusted links to place and orient the *de novo *scaffolds onto the reference genome, and then we join nearby scaffolds, provided that there is a single logical link (not necessarily trusted on its own) that goes from one scaffold to the other consistently with the placement of the scaffolds on the reference (Figure 1b).

#### Correcting misassemblies

Consider a scaffold for which part aligns to one place on the reference genome and an adjacent part aligns to another place. This could be due to an evolutionary rearrangement or to misassembly. To allow for both possibilities, we first define a window around the juncture in the scaffold, and then apply a consistency check algorithm (see Materials and methods for details) localized to the window itself (Figure 1c). If this check fails, we break the scaffold. The idea is that we do not want to run the consistency check algorithm on the whole assembly, since the regions at low coverage would yield a very large number of false positives.

#### Smoothing the assembly

Once the operations just described - that use the reference genome - have been run, a series of *de novo *assembly operations can be carried out, without using the reference genome. These operations move reads to better homes within the assembly, join contigs when possible, break contigs where needed, and so forth.

## Results

### Validation of the assisted assembly algorithm

We tested the performance and accuracy of our assisted assembly algorithms against the 7.6× high quality draft assembly of *C. familiaris *[3]. To do that, we first randomly selected whole plates from the original data set up to twofold coverage on high-quality bases (Q20, per-base error rate = 1%). With this 2× data set we performed a *de novo *assembly followed by an assisted assembly against the human genome (build 36), which has an average divergence from dog of 0.35 substitutions per site. The assisted assembly had a 7% net increase in reads assembled, an 8% improvement of total contig length, and an almost threefold improvement of scaffold length (Table 1).

**Table 1:** Comparison between initial, assisted, and theoretical 2× canine assemblies

	***Canis familiaris *- 2× assembly**		
	
	**Initial draft**	**Assisted**	**Theoretical**
Bases assembled (%)	81.0	86.5	94.1
Total contig length (Mb)	1,697	1,823	1,969
N50 contig (kb)	2.5	2.8	3.3
N50 scaffold gapped (kb)	18.6	53.1	4,039.7
N50 scaffold ungapped (kb)	10.3	36.8	3,519.1

In parallel, we generated a 'theoretical 2× assembly' by taking as input the high quality draft assembly and removing all the reads that were not present in the randomly selected set used to generate the canine 2× assembly. This represents a theoretical upper limit assembly - that is, the ideal best possible assembly for the 2× data set. Comparison of the real and theoretical 2× assemblies shows that the assisted assembly greatly improves the initial *de novo *assembly in terms of genomic content: total contig length in the initial assembly is 1.70 Gb, which improves to 1.82 Gb after assist, versus 1.97 Gb of total contig length in the theoretical assembly. Assisted assembly also dramatically improves the N50 (length-weighted median) scaffold length (from 18.6 kb to 53.1 kb), but does not reach the theoretical limit (4.0 Mb). The large discrepancy between assisted and theoretical scaffold length is largely due to the fact that 'holes' in the assembly - that is, regions that were not recovered by the assisting algorithm - greatly increased fragmentation at the scaffolding level.

**Figure 2:**
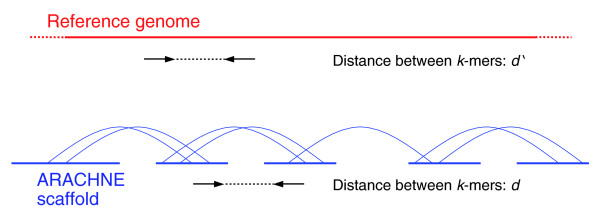
**Validation test**. From the target assembly, we randomly select a pair of high-quality k-mers at distance d from each other. The pair is declared valid if the two k-mers are both present in the reference genome, with the same orientation and a separation d', approximately equal to d. This operation is repeated for many pairs. We report the fraction of such pairs that are valid.

We then devised the following statistical validation test to determine the quality of any given assembly against a finished or high quality draft assembly. We randomly selected a large number of high quality oriented k-mers from the 2× assembly (in practice, we used k = 24), and then we ascertained the frequency at which k-mers at distance d from each other in the 2× assembly (for various values of d) appeared to be misassembled with respect to the high quality draft (Figure 2, Table 2).

**Table 2:** Accuracy of initial and assisted assemblies, estimated using the Assembly proximity test*

	**1 kb**	**2 kb**	**6 kb**	**10 kb**	**20 kb**	**60 kb**	**100 kb**
Initial draft	97.9%	97.5%	97.4%	97.1%	96.2%	95.3%	94.4%

Assisted	98.2%	98.1%	98.1%	98.0%	98.0%	97.9%	97.9%

*Random paired k-mers were selected from the 2× canine assemblies and then matched against the high quality draft assembly. The table shows the success rate for various values of d (the distance between the pairs).

We applied the validation test to the *de novo *and the assisted assemblies of *C. familiaris *(we could not apply the test to the other assemblies, since it requires a finished or high quality draft assembly to use as the 'truth'). We found that the assembly after assist is the most accurate of the two, notwithstanding the fact that scaffolds are much longer in the assisted version. For example, the fraction of pairs of *k*-mers 100 kb apart that were confirmed by the high quality assembly was 94.4% in the initial 2× draft and 97.9% in the 2× assisted assembly.

### 2× mammalian assemblies

A major application for the assisted assembly algorithm is the 2× mammalian genomes sequenced for annotation of the human genome [5,9]. To date, 21 2× assemblies have been generated using these algorithms, with human and dog as references. One of these, the assembly of the cat genome, has also been mapped to the chromosomes using an existing radiation hybrid map [7].

These reference genomes were selected based on their high genome quality, their positions in two different groups of the eutherian tree, and their relatively low divergence from the common ancestor of mammals. The mouse genome, although more complete than the dog, was not used as a reference genome because of its high divergence rate.

The assist process had a clear effect on all the original 2× mammalian assemblies (see Materials and methods): read usage and total contig length improved, on average, about 10%; N50 contig length increased, on average, from 2.8 kb to 3.0 kb; and scaffold N50 size increased by up to a factor of 5. Table 3 shows data from four examples that were assembled with the exact same version of the code. As expected, the impact of the assisting procedure is larger when the branching length between the assisted genome and the reference genome is shorter: after assist, for example, the N50 scaffold length for bushbaby, *Otolemur garnetti*, was approximately 72 kb, almost twice the N50 scaffold length of the elephant, *Loxodonta Africana *(Table 3).

**Table 3:** Assembly statistics for initial drafts and assisted assemblies for a selection of 2× mammal assemblies

	**Four projects from Mammal24 - 2× assemblies**							
	
	***Otolemur garnetti *(bushbaby)**		***Loxodonta africana *(African elephant)**		***Oryctolagus cuniculus *(rabbit)**		***Cavia porcellus *(guinea pig)**	
				
	**Initial**	Assisted*	**Initial**	Assisted*	**Initial**	Assisted*	**Initial**	Assisted*
Bases assembled (%)	76.1	85.7	77.5	84.2	80.1	85.3	75.6	82.4
Total contig length (Mb)	1,672	1,905	2,089	2,314	1,925	2,080	1,658	1,853
N50 contig (kb)	2.6	2.9	2.7	2.7	2.7	2.9	2.5	2.6
N50 scaffold gapped (kb)	13.6	71.6	11.8	37.0	13.3	53.9	11.0	44.5
N50 scaffold ungapped (kb)	9.1	37.6	8.4	15.9	9.5	20.1	7.6	12.2

*All assemblies were assisted against two references, *Homo sapiens *and *C. familiaris*.

### Assisting high coverage data sets with cloning bias

In theory, the assisted assembly should work equally well to rescue genomes with severe cloning bias resulting in low coverage sequence in certain portions of the genome. We therefore applied the same algorithms on the malaria strain *P. falciparum *HB3. It was sequenced to 8× [8], but the resulting assembly had surprisingly low connectivity and shorter-than-expected total contig length. In fact, cloning bias reduced the coverage to 2× or less for about 20% out of the 24 Mb genome, which is considerably more than the 0.03% expected for an average 8× assembly.

The reference strain *P. falciparum *3D7 was used as a reference [10]. This is of almost finished quality, and is 0.12 substitutions per site diverged from the HB3 strain [8]. The assisting process recovered almost 4 Mb of low coverage regions (17% of the genome), while the N50 scaffold length increased by almost a factor of three (Table 4).

**Table 4:** Assembly statistics for initial drafts and assisted assemblies for the 8× assembly of *P. falciparum *HB3, which has severe cloning bias

	***P. falciparum *HB3 - 8× assembly**	
	
	**Initial draft**	**Assisted**
Bases assembled (%)	85.6	93.4
Total contig length (Mb)	19.8	23.5
N50 contig (kb)	13.7	15.4
N50 scaffold gapped (kb)	17.0	48.8
N50 scaffold ungapped (kb)	16.8	47.5

## Discussion

We show that the assisted assembly process significantly improves contiguity and quality of low coverage mammalian assemblies and that it can be successfully applied to genomes with locally low coverage caused by cloning bias, such as *P. falciparum *HB3 [8]. While some previous work has described the use of information such as optical maps or draft assemblies of the same species to inform the assembly process [11-13], we believe that the algorithms described here stand out, as they carefully use the conserved synteny information of reads aligned to a reference genome to leverage information already existing within a the target genome sequence data.

The choice of reference genome(s) is critical when performing assisted assembly. Clearly, using a closely related genome to improve an initial draft assembly will have a bigger impact on the final draft assembly, and the accuracy and completeness of a reference genome also contribute. In the assemblies we generated, the number of validated pairs aligning uniquely to the reference varied from 18.5% of the alignments of the guinea pig against the human reference, to 74.3% of the alignments of strain HB3 of *Plasmodium *against the reference strain 3D7 (Table 5).

**Table 5:** Statistics of the alignments of reads onto the reference genomes

	**Assisted on**	**Reads aligning target uniquely**	**Valid pairs aligning target uniquely**
*Plasmodium falciparum *HB3	*Plasmodium falciparum *3D7	79.1%	74.3%
*Canis familiaris *- 2× assembly	*Homo sapiens*	64.1%	35.1%
*Loxodonta africana*	*Homo sapiens*	51.1%	22.7%
*Oryctolagus cuniculus*	*Homo sapiens*	55.3%	25.2%
*Otolemur garnetti*	*Homo sapiens*	68.8%	38.0%
*Cavia porcellus*	*Homo sapiens*	47.8%	18.5%
*Loxodonta africana*	*Canis familiaris*	49.3%	28.8%
*Oryctolagus cuniculus*	*Canis familiaris*	48.8%	29.8%
*Otolemur garnetti*	*Canis familiaris*	59.6%	43.9%
*Cavia porcellus*	*Canis familiaris*	41.6%	22.4%

The projects from the Mammal24 set were assisted against both human and canine references.

Still, the most critical factor is the ability to uniquely align target reads to the reference genome. The BLASTZ algorithm [6] aligns reads reliably when the genomes are up to approximately 0.45 substitutions per site apart, as was determined as a prerequisite for the project to annotate the human genome using 24 low coverage mammals [5].

Many of the parameters that affect the accuracy of the read to reference genome alignments are generally less favorable for new sequencing technologies, where short reads with higher error rate are more common. This means that the current methodology can only be used on really closely related species using new short-read sequence technologies.

## Materials and methods

### Code and assembly

We used ARACHNE [14,15] to generate initial draft assemblies, and all the assisted assembly tools were developed inside the framework provided by ARACHNE. The code is available for download from [16], as well as the assemblies generated for this paper, together with the set of 'lab notes' used to generate the assemblies. All the assemblies reported in Table 2 were generated with the same frozen code. The original set of 21 projects in Mammal24 is publicly available from [17].

### Placing reads on a reference genome

We used the aligner BLASTZ [6] with default arguments to align the 2× mammalian assemblies against both human and canine references. At the end of the process we filtered the alignments from BLASTZ by discarding those with an alignment score lower than a given threshold (3,000), hence allowing for a read to be multiply placed.

We used the aligner QueryLookupTable with parameters MF = 5000 SH = True MC = 0.15 to align the WGS reads of *P. falciparum *HB3 against the strain 3D7. The aligner is part of the standard distribution of the ARACHNE code and is distributed together with the assisting code.

### Enlarging contigs

The process of enlarging contigs consists of allowing groups of reads that appear to overlap based on their position on the reference to extend existing *de novo *contigs. This is realized in practice as an assisted improvement of the layout code: reads that are adjacent to each other in their group on the reference are tested for read-read alignment, and if a read-read alignment exists, this is used to seed the positioning of the new read onto the existing layout (hence extending the layout of the contig). After assisted layout, the *de novo *consensus module is called with standard arguments.

### Joining scaffolds

Scaffolds are anchored to the reference genome by using the set of pairs that align uniquely and validly onto the reference genome. A pair aligning uniquely onto the reference genome is called a 'validated pair' if the absolute value of its stretch (defined as the difference between observed separation and given separation divided by the given standard deviation) does not exceed 5. The end reads of validated pairs are called 'validated reads'.

For a given scaffold, we look at all the validated reads: each of these reads implicitly maps and orients the scaffold on the reference genome. We then sort the validated reads by their start on the scaffold. Two adjacent validated reads are defined to be 'consistent' if they map and orient the scaffold on the same reference sequence, and if the absolute rate of the compression rate c (that is, the ratio between the distance of the two reads on the scaffold and on the reference genome) is such that 1/3 < c < 3.

A scaffold is anchored to the reference genome if there are at least two validated reads in the scaffold, and if all the pairs of consecutive validated reads in the scaffold are consistent. In practice, we found that most scaffolds contain at least a few validated reads, even when only a fraction of the reads was actually validated.

### Correcting misassemblies

We now focus on scaffolds for which the following happens: the scaffold contains several validated reads (which are sorted by their start on the scaffold), and the validated reads are divided in two 'clean' sets - that is, there is one, and only one, non-consistent pair of consecutive validated reads, say r1 and r2. We then define a window of possible misassembly as the interval [a, b), where a is the start on the scaffold of r1, and b the end on the scaffold of the read r2.

We then apply the following consistency check to the window of possible misassembly: if there exists a point in the window with read coverage <3 and no insert coverage, then the contig is broken at the juncture, and eventually the scaffold is broken in its connected components. In other words, the contig is broken if at any point the window is 'held together' by a single read-read overlap.

### Validation: assembly proximity test

This section defines what a 'valid' pair of k-mers is, for the proximity validation test. We start by fixing a target assembly (for example, one of the 2× dog assemblies) together with a reference finished grade assembly of the same species (for example, the full coverage draft assembly of dog).

We then randomly select from the target assembly a high quality pair of oriented k-mers at distance d from each other. This is defined as a pair of k-mers, such that: all the bases in the two k-mers have quality 50; and the separation between the two k-mers is d. Next, we define the standard deviation of such a pair. If the two k-mers belong to the same contig, then this is defined as the maximum between k and d/100. Otherwise, the square of the standard deviation of the pair is defined as the sum of the squares of the standard deviations of the gaps between the two contigs containing the k-mers.

We now look for the pair in the reference assembly. The pair is 'valid' if we can find at least one instance of the pair onto the reference assembly, such that: the relative orientation of the two k-mers in the pair is the same as in the target assembly; and the stretch of the pair does not exceed 3, where stretch is defined as (d' - d)/stdev, where d' is the distance between the k-mers on the reference, and stdev the standard deviation of the pair defined above.

## Abbreviations

N50: length-weighted median; WGS: whole-genome shotgun.

## Authors' contributions

ESL and KLT proposed the assisted assembly concept. SG carried out the research and wrote the code. DBJ proposed the validation methodology. SG, DBJ and KLT wrote the paper. All authors read and approved the final manuscript.
